# Supplementary material for: MOGAT: A Multi-Omics Integration Framework Using Graph Attention Networks for Cancer Subtype Prediction
Source: Int J Mol Sci. 2024 Feb 28;25(5):2788. doi: 10.3390/ijms25052788 (PMC10932030; doi:10.3390/ijms25052788)
Supplement: Supplementary file 1 [file ijms-25-02788-s001.zip › Supplementary Material S1_Clinical Features Preprocessing.pdf]

Supplementary Material S1: Clinical Features Preprocessing

Table S1: Clinical Features of TCGA-BRCA after the one-hot vector conversion. For example. Menopause has four statuses- Indeterminate, Peri, Post, and Pre, which became binary variables after one-hot vector conversion. Each variable except the last row, age, is categorical. In total, there are 31 clinical features. Gower Metric was used to construct the patient similarity network for this dataset.

**Clinical Features of TCGA-BRCA**
menopause_status_Indeterminate (neither Pre or Postmenopausal)
menopause_status_Peri (6-12 months since last menstrual period)
menopause_status_Post (prior bilateral ovariectomy OR >12 mo since LMP with no prior hysterectomy)
menopause_status_Pre (<6 months since LMP AND no prior bilateral ovariectomy AND not on estrogen replacement)
race.demographic_american indian or alaska native
race.demographic_asian
race.demographic_black or african american
race.demographic_not reported
race.demographic_white
tumor_stage.diagnoses_not reported
tumor_stage.diagnoses_stage i
tumor_stage.diagnoses_stage ia
tumor_stage.diagnoses_stage ib
tumor_stage.diagnoses_stage ii
tumor_stage.diagnoses_stage iia
tumor_stage.diagnoses_stage iib
tumor_stage.diagnoses_stage iii
tumor_stage.diagnoses_stage iiia
tumor_stage.diagnoses_stage iiib
tumor_stage.diagnoses_stage iiic
tumor_stage.diagnoses_stage iv
tumor_stage.diagnoses_stage x
metastatic_breast_carcinoma_estrogen_receptor_status_Negative
metastatic_breast_carcinoma_estrogen_receptor_status_Positive
metastatic_breast_carcinoma_progesterone_receptor_status_Negative
metastatic_breast_carcinoma_progesterone_receptor_status_Positive
metastatic_breast_carcinoma_lab_proc_her2_neu_immunohistochemistry_receptor_status_Equivocal
metastatic_breast_carcinoma_lab_proc_her2_neu_immunohistochemistry_receptor_status_Negative
person_neoplasm_cancer_status_TUMOR FREE
person_neoplasm_cancer_status_WITH TUMOR
age_at_initial_pathologic_diagnosis

Table S2: Clinical Features of METABRIC after the one-hot vector conversion. There are 14 clinical features. Each variable except age is categorical. Gower Metric was used to construct the patient similarity network for this dataset.

Clinical Features
age
menopausal_status_inferred_post
menopausal_status_inferred_pre
ER.Expr_+
ER.Expr_-
Her2.Expr_+
Her2.Expr_-
PR.Expr_+
PR.Expr_-
Ethnicity.Call_African/European
Ethnicity.Call_Asian
Ethnicity.Call_European
Ethnicity.Call_European/Asian
Ethnicity.Call_OddCluster
