# Supplementary material for: MOGAT: A Multi-Omics Integration Framework Using Graph Attention Networks for Cancer Subtype Prediction
Source: Int J Mol Sci. 2024 Feb 28;25(5):2788. doi: 10.3390/ijms25052788 (PMC10932030; doi:10.3390/ijms25052788)
Supplement: Supplementary file 1 [file ijms-25-02788-s001.zip › Supplementary Material S2_Copy Number Alteration Data Preprocessing.pdf]

Supplementary Material S2: Copy Number Alteration Data Preprocessing

Definition

Copy number variation (CNV) refers to a circumstance in which the number of copies of a specific DNA segment varies among individuals' genomes. The individual variants may be short or include thousands of bases. These structural differences may have come about through duplications, deletions, or other changes and can affect long stretches of DNA. Such regions may or may not contain a gene(s).

Raw Data

Copy number variation data were downloaded from the Genomic Data Commons (GDC) portal (https://portal.gdc.cancer.gov/) for the TCGA-BRCA cohort. The data was acquired from whole exome sequencing experiments and contained information about each patient's copy number of DNA segments across the genome. Each file contained copy number information for each patient – the chromosome number, start and end position of the genomic coordinate, the number of probes, and segment mean which is $\log_2(CopyNumber/2)$. The number of rows was different for each sample. A snapshot of the file is given in Figure 1.

ID	chrom	loc.start	loc.end	num.mark	seg.mean
f4aa579c-fa0c-4313-a4a1-ca88a367d71f	1	62920	15823420	8321	0.0264
f4aa579c-fa0c-4313-a4a1-ca88a367d71f	1	15827002	15827430	6	-1.9250
f4aa579c-fa0c-4313-a4a1-ca88a367d71f	1	15827706	16542868	302	0.0222
f4aa579c-fa0c-4313-a4a1-ca88a367d71f	1	16544783	16617312	10	-0.8436
f4aa579c-fa0c-4313-a4a1-ca88a367d71f	1	16617327	16864367	48	-0.2007
f4aa579c-fa0c-4313-a4a1-ca88a367d71f	1	16868660	16898730	12	-0.9755

Figure S1: Snapshot of the data file for copy number variation for a single patient. The rows contain the sample ID, chromosome number, genomic location of start and end points, number of probes used, and segment mean.

Conversion to Gene-Centric Matrix

To analyze the data, the individual patient datasets were combined into a single dataset containing 1,268,168 rows. CNTools [1], a software tool for analyzing copy number data, was then used to convert the raw copy number data into a gene-centric matrix. This matrix contained the average copy number of each gene across the patient group and allowed for identifying genes frequently amplified or deleted in cancer. The gene-centric matrix was used as the basis for further analyses. A small example of this conversion is given below.

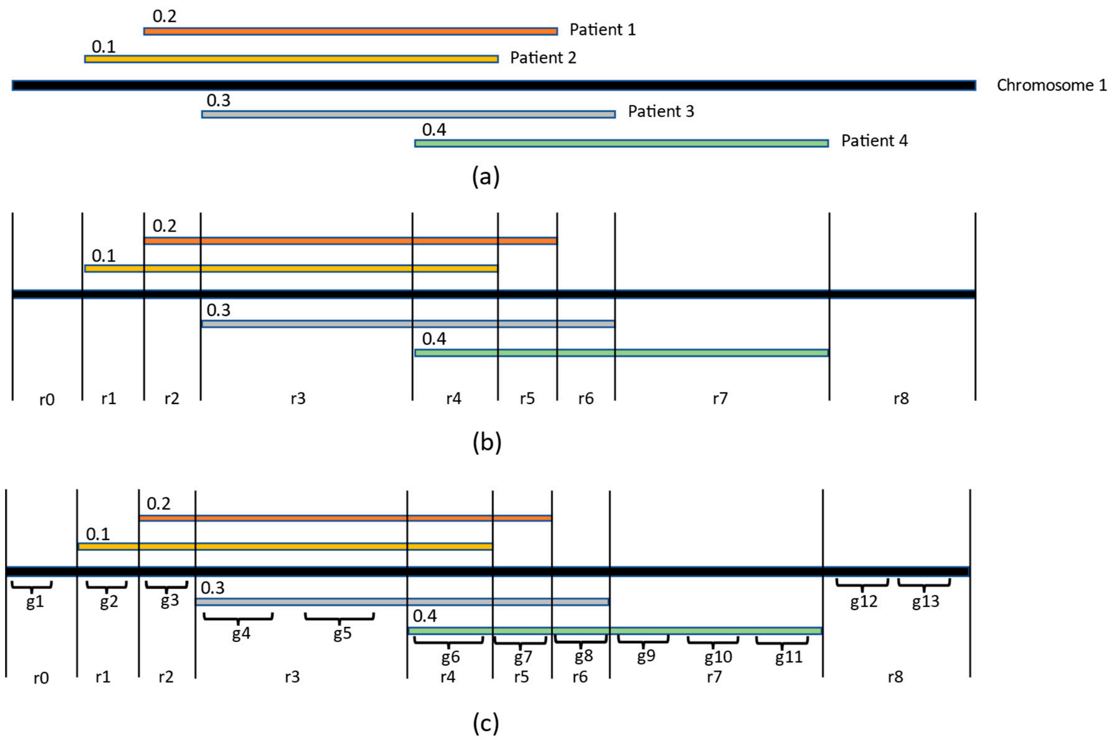

Figure S2: An example of copy number variations and converting it into region by sample matrix to gene-centric matrix. (a) Four patients' copy number alteration data in segment mean according to genomic coordinates on chromosome 1; (b) the genomic locations are divided into smaller regions; (c) genes in those regions are marked.

Suppose the merged file contains information on copy number variation for four patients, Patients 1 to 4, on chromosome 1. This information is color-coded as orange, yellow, gray, and green and includes the value of segment mean, calculated as log2(copy-number/2). The chromosome regions are divided into r0 to r8 based on whether they are shared by one or more patients or none. For instance, r0 regions are not shared by any patients, while r1 is shared by Patient 1 only, and r2 is shared by Patient 1 and Patient 2. Using this information, a region by sample matrix is constructed, showing the copy number variation for each patient in each region, as shown in Table 1.

Table S1: Region by Sample Matrix for Example Data.

Regions	Patient 1	Patient 2	Patient 3	Patient 4
r0	0	0	0	0
r1	0	0.1	0	0
r2	0.2	0.1	0	0
r3	0.2	0.1	0.3	0
r4	0.2	0.1	0.3	0.4
r5	0.2	0	0.3	0.4
r6	0	0	0.3	0.4
r7	0	0	0	0.4
r8	0	0	0	0

The next step involves transforming the region by sample matrix into a gene-centric matrix, as shown in Table S2, by determining which genes have genomic coordinates corresponding to each region. In this case, genes that fall within a given region will have the same segment mean value as their assigned region.

Table S2: Gene-Centric Matrix for Example Data.

Genes	Patient 1	Patient 2	Patient 3	Patient 4
g1	0	0	0	0
g2	0	0.1	0	0
g3	0.2	0.1	0	0
g4	0.2	0.1	0.3	0
g5	0.2	0.1	0.3	0
g6	0.2	0.1	0.3	0.4
g7	0.2	0	0.3	0.4
g8	0	0	0.3	0.4
g9	0	0	0	0.4
g10	0	0	0	0.4
g11	0	0	0	0.4
g12	0	0	0	0
g13	0	0	0	0

Patient Similarity Matrix Construction

The gene-centric copy number variation (CNV) data was used to construct a patient similarity matrix. The similarity matrix was generated using Pearson's correlation coefficient, which measures the strength and direction of the linear relationship between two variables. Then, the similarity network was constructed by taking the top 3 most similar patients based on Pearson’s correlation coefficient as edges for each patient.

References

[1] J. Zhang, “CNTools: Convert segment data into a region by sample matrix to allow for other high level computational analyses.” 2019.
