# Supplementary material for: MOGAT: A Multi-Omics Integration Framework Using Graph Attention Networks for Cancer Subtype Prediction
Source: Int J Mol Sci. 2024 Feb 28;25(5):2788. doi: 10.3390/ijms25052788 (PMC10932030; doi:10.3390/ijms25052788)
Supplement: Supplementary file 1 [file ijms-25-02788-s001.zip › Supplementary Material S3_Coexpression Features Extraction.pdf]

Supplementary Material S3: Coexpression Features Extraction Using WGCNA

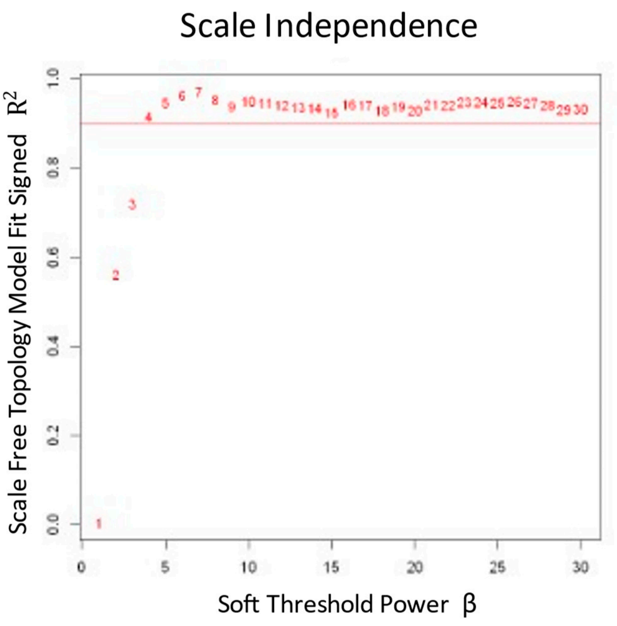

Figure S1: Scale Free Topology Model Fit R^2 Vs Soft Threshold β for TCGA-BRCA.

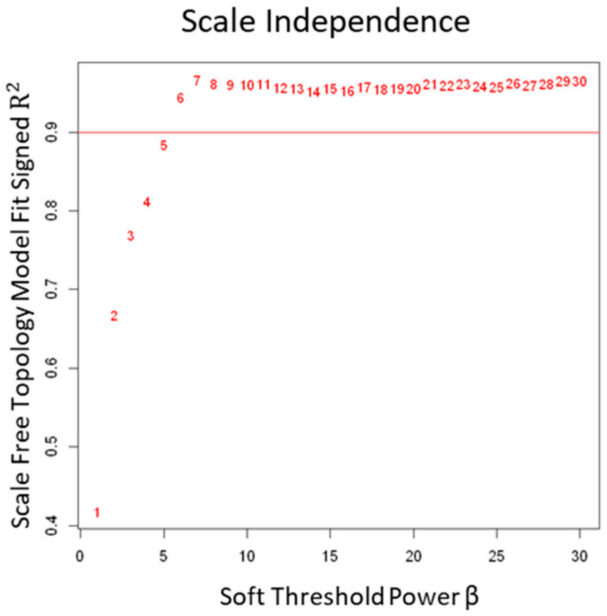

Figure S2: Scale Free Topology Model Fit R^2 Vs Soft Threshold β for METABRIC.

Table S1: Size of the modules in terms of number of genes for TCGA-BRCA.

Module Index	# of Genes	Module Index	# of Genes
0	5581	20	114
1	2244	21	113
2	1640	22	109
3	1483	23	105
4	1444	24	101
5	1066	25	98
6	939	26	91
7	928	27	85
8	709	28	83
9	336	29	82
10	317	30	80
11	261	31	75
12	257	32	70
13	251	33	64
14	197	34	63
15	187	35	62
16	144	36	59
17	139	37	53
18	122	38	52
19	116	39	42

Table S2: Size of the modules in terms of number of genes for METABRIC.

Module Index	# of Genes	Module Index	# of Genes	Module Index	# of Genes	Module Index	# of Genes	Module Index	# of Genes	Module Index	# of Genes	Module Index	# of Genes
0	10278	7	454	14	101	21	60	28	35	35	21	42	16
1	3124	8	413	15	87	22	54	29	35	36	21	43	16
2	1241	9	263	16	85	23	54	30	33	37	18	44	15
3	1025	10	203	17	69	24	53	31	30	38	18	45	14
4	740	11	165	18	69	25	52	32	25	39	17	46	13
5	632	12	134	19	67	26	42	33	24	40	17	47	12
6	497	13	108	20	63	27	38	34	24	41	17	48	11
